# Exploring medical students' experience of the learning environment: a mixed methods study in Saudi medical college

**DOI:** 10.1186/s12909-024-05716-4

**Published:** 2024-07-03

**Authors:** Mohammed Almansour, Noura Abouammoh, Reem Bin Idris, Omar Abdullatif Alsuliman, Renad Abdulrahman Alhomaidi, Mohammed Hamad Alhumud, Hani A. Alghamdi

**Affiliations:** 1 Department of Medical Education, College of Medicine, King Saud University, Riyadh, Saudi Arabia; 2 Department of Family and Community Medicine, College of Medicine, King Saud University, Riyadh, Saudi Arabia; 3 College of Medicine, King Saud University, Riyadh, Saudi Arabia

**Keywords:** Learning environment, Medical education, Comparative studies, Mixed methods

## Abstract

**Background:**

In medical education, the learning environment (LE) significantly impacts students' professionalism and academic performance. Positive LE perceptions are linked to better academic outcomes. Our study, which was conducted 15 years after curriculum reform at King Saud University's College of Medicine, aimed to explore students' perspectives on their LE and identify areas for improvement. By understanding their experiences, we strive to enhance LE and promote academic success.

**Methods:**

This mixed-method study employed an explanatory sequential approach in which a cross-sectional analytical survey phase was collected first using the Johns Hopkins Learning Environment Scale (JHLES), followed by qualitative focus groups. Findings from quantitative and qualitative methods were integrated using joint display.

**Results:**

A total of 653 medical students completed the JHLES. The total average score was 81 out of 140 (16.8), and the average subscale scores ranged from 2.27 (0.95) for inclusion and safety to 3.37 (0.91) for community of peers. The qualitative approach encompasses both inductive and deductive analyses, identifying overarching themes comprising proudness, high expectations and competition, and views about the curriculum. The integration of results emphasizes the need for continued efforts to create a supportive and inclusive LE that positively influences students' experiences and academic success.

**Conclusion:**

This research offers valuable insights for educational institutions seeking to enhance medical education quality and support systems. Recommendations include faculty development, the cultivation of supportive environments, curriculum revision, improved mentorship programs, and initiatives to promote inclusivity and gender equity. Future research should explore longitudinal and comparative studies, innovative mixed methods approaches, and interventions to further optimize medical education experiences. Overall, this study contributes to the ongoing dialog on medical education, offering a nuanced understanding of the complex factors influencing students' perceptions and suggesting actionable strategies for improvement.

## Background

The learning environment of medical students plays a significant role in shaping qualified, well-rounded physicians. It can also impact students' professionalism, ethics, and morals. As these students graduate and begin their professional practice, their competency can be a direct reflection of the medical institutes from which they graduated. The learning environment (LE) is a term used to describe the physical, cultural, and psychosocial climate in which learning takes place [1]. Students' skills, knowledge, and attitudes are influenced by the teaching and learning environment of their educational institutes. The interactions they have with their peers, faculty members, and administrators play a role in their learning environment. The curriculum that is taught to students is part of this environment, and the curriculum's design is a vital component [2].

The impact of LE on the academic performance of medical students is significant. Therefore, it is crucial to provide a supportive environment that positively influences students' perceptions of their LE. Research has consistently shown that students who perceive their LE to be positive and supportive are more likely to perform well academically [3]. Conversely, students who perceive their LE to be negative may experience adverse effects on their academic performance [3].

A student-centered curriculum of outstanding standards must be provided, and evaluation of the educational setting at both academic and clinical sites is essential [4]. King Saud University's College of Medicine program is seven years long, starting with a preparatory year, followed by two basic sciences (preclinical) years, then three clinical-practice years, and a one-year internship. The program employs a combination of problem-based learning and interactive lecturing to teach medical and healthcare-related sciences, emphasizing critical thinking and self-directed learning. Clinical training programs provide hands-on experience, with the goal of producing skilled and compassionate healthcare professionals.

Two studies were conducted at the College of Medicine at King Saud University (COM-KSU). The first study was conducted in 2008, prior to the college's curriculum reform in 2009, which transitioned from a traditional to a system-oriented hybrid curriculum [5]. Researchers utilized the Dundee Ready Educational Environment Measure (DREEM) scale to evaluate the learning environment (LE), and the results indicated that first-year students had significantly higher scores than other students [5]. Additionally, preclinical students had significantly greater scores than did clinical students, and gender was not a statistically significant factor [5].

The second study was conducted in 2014, where fifth-year medical students were evaluated using the DREEM scale to assess their perception of the LE [6]. The study revealed that the students' perception of the educational environment was satisfactory [6].

The Johns Hopkins Learning Environment Scale (JHLES) was created by the Johns Hopkins University School of Medicine to evaluate the quality of the learning environment for residents and medical students [7]. The 28-item scale helps medical educators identify areas of improvement by assessing seven factors or subscales, comprising community of peers, faculty relationships, academic climate, meaningful engagement, mentoring, inclusion and safety, and physical space [7].

The aim of our study was to investigate the perceptions of medical students regarding their LE at the COM-KSU 15 years after the curriculum was reformed. We seek to understand the experiences of students in this particular LE and gain insights into the factors that influence their perceptions of the LE. By exploring the students' perspectives, we aim to identify areas where improvements can be made to enhance LE and ensure that it is conducive to learning and promotes academic success.

## Methods

### Aim, design, and setting

This mixed-method study aimed to investigate students’ perceptions of the LE at COM-KSU 15 year proceeding a curriculum change, followed by an exploration of their perspectives aiming to identify areas of improvement of the LE. This study employed an explanatory sequential approach in which a cross-sectional analytical survey phase collected first, followed by qualitative focus groups. The research was carried out between November 2022 and March 2023 within the College of Medicine at King Saud University (COM-KSU), which is the pioneering medical education institution in the Kingdom of Saudi Arabia and is located in the capital city of Riyadh.

## Participants and sampling

All the COM-KSU undergraduate students and interns were invited to participate in the study, with a total of 1471 students and 268 interns. The total number of enumeration techniques over the period of the study was used. Convenient sampling was employed in this study. The decision to employ convenient sampling was based on practical considerations of the accessibility and availability of participants. Consequently, a total of 653 individuals voluntarily participated in the first phase of the study, and the research team initiated the participant recruitment process by extending invitations to all undergraduate students and interns enrolled in the COM-KSU. The invitations were disseminated via multiple channels, including email, WhatsApp groups, and personal visits to each classroom within the college.

The data collection process comprised two distinct online surveys, each serving a specific purpose. The first survey focused on the quantitative phase and included questions related to demographic information and the Johns Hopkins Learning Environment Scale (JHLES). The second survey, designed for registration in the qualitative phase, included demographic inquiries along with a means of contact and the provision of available time slots. Subsequently, the research team communicated with the registered participants and arranged for focused group discussions (FGDs) to be conducted. Two FGDs were needed (5 and 7 participants) based on the theory of data saturation. Each FGD lasted approximately 70 min and was held at the College of Medicine. The discussions were facilitated by one of the authors, who is a qualitative methodologist and a faculty member at the same college, and the participants were comfortable discussing negative views as they were discussing positive views.

## Measures

In the quantitative study phase, an online survey encompassing various components was developed. This survey collected demographic data, including information on gender, age, academic year, GPA, employment status, marital status, and residence type. Additionally, the Johns Hopkins Learning Environment Scale (JHLES), a validated tool used for assessing undergraduate medical school learning environments, was used. The JHLES consists of 28 items distributed across seven domains, and its use for this study was conducted without the need for direct permission, as it is publicly available.

In the qualitative study phase, students and interns were actively engaged in Focus Group Discussions (FGDs), aimed at eliciting their perspectives on the learning environment (LE). The FGDs employed a topic guide comprising open-ended questions aligned with the LE domains delineated by the JHLES. These questions included inquiries such as "How would you characterize your relationships with your peers?" and "To what extent does the college environment support collaboration with fellow students from the same college?" Furthermore, participants were asked to share their opinions regarding the faculty and provide insights into their perceptions of the curriculum. The FGDs were complemented with probing questions and follow-up queries to delve deeper into participants' experiences and perspectives.

## Statistical analysis

For the first phase in this study, sociodemographic data were presented using descriptive statistics. The mean and standard deviation (SD) for the total score and the seven domains of the JHLES were calculated. Cross-tabulation was used to explore the relationships between the JHLES scores and the sociodemographic variables, and tests of significance through chi-square tests and ANOVA were performed. All analyses were performed using R (version 4.2.2), [8].

## Qualitative data collection

The questions in the topic guide included probing questions and encompassed domains and questions from the JHLES. As open-ended questions were used to collect data, themes included deductive and inductive analysis. Inductive analysis was based on a priori themes based on the JHLES domains.

## Qualitative analysis

Thematic analysis was adopted for qualitative analysis. This approach was proposed by Ritchie and Spencer (1994) to be helpful in providing a sequential structure for data analysis. This was conducted using NVivo software version 11.4.2. Using software increases the efficiency of data organization and retrieval. Familiarization, descriptive coding, basic analysis, and interpretation are the steps followed in the data analysis, and quotes from the participants were used to support the themes. Analyzing the data and identifying common descriptive themes were tasks shared with the team. The team agreed on a coding frame. The analysis was conducted independently, and the results are presented in comparison to the quantitative findings in Table 5.

## Mixed methods integration

Findings from quantitative and qualitative methods were integrated using joint display. The outcomes of the JHLES and FGDs were compared side-by-side. Integrating findings can create a holistic understanding of the learning environment of the College of Medicine, leading to a conclusion where the whole is greater than the sum of its parts.

Joint display of the data provided visual means of presenting qualitative and quantitative findings granting the ability to associate reasoning with different item score. Qualitative findings complement the quantitative findings in providing meaning to the score and explored in students’ perspective reasons for these scores. The qualitative findings also explained how students’ pride and perception about their own status reflect on the JHLES score. Students’ needs and preferences were expressed explicitly during the FGDs.

### Ethical considerations

This study was approved by King Saud University’s Institutional Review Board (KSU IRB) with the approval number E-22–7298. Electronic informed consent was obtained from all participants in the quantitative arm, and written informed consent was obtained from all participants in the qualitative arm prior to their participation in the study.

## Results

### Quantitative results

#### Sociodemographic characteristics

Table 1 presents the sociodemographic characteristics of all participants. The total number of medical students and interns included in this phase of the study was 653. Of those studied, there was an almost equal gender distribution, with males making up slightly more than half (59%). There were relatively varied numbers of academic years, with less than average representation coming from the intern level at a participation rate of only 4%, while the highest engagement occurred during fourth-year studies at approximately 26%. Most individuals boasted high academic records, achieving an above-average GPA of 4.50–5.00 (65.7%). Of those who participated, a small fraction had lower grades below a GPA of 4 (11.5%). The majority of the participants were unemployed (96.2%), while less than 4% were either employed (full- or part-time) or freelancers (1.5%). Regarding personal life traits, most of the participants were single (98.5%) and lived with their families residing in Riyadh (93%).

**Table 1:** Sociodemographic characteristics of the participants

**Characteristics**		**Total (%)** 653 (100%)
Gender	Male	385 (59)
	Female	268 (41)
Academic Year	1^st^year	116 (17.8)
	2nd year	114 (17.5)
	3rd year	137 (21)
	4th year	169 (25.9)
	5th year	90 (13.8)
	Intern	27 (4.1)
GPA	4.50–5.00	429 (65.7)
	4.00–4.49	149 (22.8)
	Less than 4.00	75 (11.5)
Employment status	Unemployed	628 (96.2)
	Employed	15 (2.3)
	Freelancer	10 (1.5)
Marital status	Single	643 (98.5)
	Other	10 (1.5)
Residency	With family	607 (93)
	Private	46 (7)
Family living in Riyadh	Yes	609 (93.3)
	No	44 (6.7)

As shown in Table 2, the overall mean score for student experience was 81 (*SD* = 16.76). Among the specific subscales, the highest mean score was observed for physical space (3.52; *SD* = 0.95), and the lowest mean score was found for inclusion and safety (2.27; *SD* = 0.95).

**Table 2:** Means and SDs for the JHLES

JHEL Scale	Mean (*SD*)
Overall score	81 (16.76)
Community of peers	3.37 (0.91)
Faculty relationships	2.74 (0.89)
Academic climate	2.88 (0.85)
Meaningful engagement	2.68 (0.94)
Mentoring	2.68 (1.15)
Inclusion and safety	2.27 (0.95)
Physical space	3.52 (0.95)

### Sociodemographic variables and overall and domain scores of the JHLES (mean and SD)

Associations between sociodemographic variables and the different domains of the JHLES as well as the overall score are represented in Table 3. Male students reported a higher mean overall score than females did (83.4 ± 17.1 and 77.5 ± 15.7, respectively). As the number of academic years increased, the first-year students reported a greater average score than did the senior-year students, with a mean overall score for first-year medical students of 87.6 (*SD* = 16.9), whereas the average score for senior-year students (fifth-year) was 74.8 ± 18.2. Students who possessed higher GPAs (4.50–5.00) achieved the highest mean score of 82.2 ± 16, while those with GPAs less than 4.00 reported the lowest average score of 73.3 (*SD* = 15). Employment status was another variable impacting students' individual perceptions of this survey total score, where employed students generally outperformed unemployed students, with higher scores (88.6 ± 18.6) than unemployed students (80.7 ± 16.7). A significant association was observed between the overall JHELS score and gender, academic year, and GPA at the 0.05 level.

**Table 3:** Associations between sociodemographic characteristics and overall and domain scores of the JHLES (mean and SD)

Student characteristics	Community of peers	Faculty relationships	Academic climate	Meaningful engagement	Mentoring	Inclusion and safety	Physical space	Overall score
**Gender**	< 0.001*	< 0.001*	0.006*	< 0.001*	0.06	0.15	0.12	< 0.001*
Male	3.5 (0.88)	2.87 (0.90)	2.95 (0.85)	2.79 (0.98)	2.75 (1.15)	2.22 (0.92)	3.57 (0.95)	83.4 (17.1)
Female	3.18 (0.92)	2.57 (0.85)	2.77 (0.83)	2.54 (0.87)	2.58 (1.14)	2.33 (0.99)	3.45 (0.94)	77.5 (15.7)
**Academic Year**	< 0.001*	< 0.001*	< 0.001*	< 0.001*	0.01*	< 0.001*	0.33	< 0.001*
1st year	3.64 (0.88)	3.18 (0.85)	3.17 (0.83)	3.30 (0.91)	2.50 (1.10)	1.82 (0.90)	3.59 (1.04)	87.6 (16.9)
2nd year	3.39 (0.99)	2.87 (0.94)	3.01 (0.84)	2.76 (0.92)	2.64 (1.13)	2.08 (0.89)	3.51 (1.04)	82.2 (18)
3rd year	3.17 (0.90)	2.52 (0.77)	2.68 (0.79)	2.51 (0.86)	2.71 (1.14)	2.38 (0.88)	3.53 (0.91)	77.2 (15)
4th year	3.47 (0.77)	2.77 (0.79)	2.93 (0.78)	2.66 (0.83)	2.82 (1.13)	2.39 (0.89)	3.58 (0.83)	82.7 (14.8)
5th year	3.10 (0.98)	2.44 (0.95)	2.61 (0.92)	2.26 (0.94)	2.49 (1.24)	2.57 (1.07)	3.42 (0.93)	74.8 (18.2)
Intern	3.43 (0.87)	2.34 (0.91)	2.60 (0.89)	2.18 (0.95)	3.24 (1.06)	2.55 (0.90)	3.19 (1.05)	76.9 (13.7)
**GPA**	< 0.001*	0.005*	0.001*	0.26	0.10	0.55	0.39	< 0.001*
4.50–5.00	3.47 (0.88)	2.80 (0.87)	2.95 (0.80)	2.69 (0.93)	2.69 (1.13)	2.24 (0.93)	3.54 (0.95)	82.2 (16)
4.00–4.49	3.33 (0.90)	2.75 (0.92)	2.83 (0.89)	2.74 (1.01)	2.79 (1.18)	2.32 (1.00)	3.55 (0.94)	81.2 (18.3)
Less than 4.00	2.86 (0.93)	2.43 (0.91)	2.56 (0.93)	2.53 (0.91)	2.44 (1.20)	2.33 (0.97)	3.38 (0.91)	73.3 (15.9)
**Employment status**	0.27	0.15	0.05	0.06	**0.02**	0.18	0.89	0.05
Unemployed	3.36 (0.91)	2.73 (0.89)	2.88 (0.84)	2.67 (0.94)	2.66 (1.15)	2.25 (0.95)	3.52 (0.95)	80.7 (16.7)
Employed	3.70 (0.63)	3.12 (0.96)	3.20 (0.99)	3.23 (0.93)	3.23 (1.28)	2.49 (1.20)	3.47 (0.79)	90.7 (18.2)
Freelancer	3.58 (0.88)	3.02 (0.75)	2.36 (0.61)	2.90 (1.27)	3.40 (0.94)	2.73 (0.78)	3.65 (1.13)	85.3 (15.7)
**Marital status**	0.52	0.92	0.78	0.71	0.08	0.09	0.05	0.55
Single	3.37 (0.91)	2.74 (0.89)	2.87 (0.85)	2.68 (0.94)	2.67 (1.15)	2.26 (0.95)	3.53 (0.95)	80.9 (16.7)
Other	3.53 (0.78)	2.72 (0.92)	2.94 (0.72)	2.80 (0.96)	3.35 (1.11)	2.83 (0.96)	3.00 (0.75)	84.6 (18.6)
**Residency**	0.25	0.43	0.36	0.84	0.97	0.98	0.27	0.81
With family	3.38 (0.91)	2.74 (0.89)	2.87 (0.84)	2.68 (0.94)	2.68 (1.15)	2.27 (0.95)	3.51 (0.95)	80.9 (16.7)
Private	3.21 (1.00)	2.84 (0.89)	2.99 (0.88)	2.71 (0.94)	2.67 (1.14)	2.27 (0.91)	3.66 (0.90)	81.6 (18)
**Family living in Riyadh**	0.16	0.75	0.73	0.81	0.51	0.87	0.10	0.46
Yes	3.39 (0.89)	2.75 (0.89)	2.88 (0.83)	2.69 (0.94)	2.69 (1.15)	2.26 (0.95)	3.52 (0.94)	81.1 (16.4)
No	3.13 (1.15)	2.70 (0.95)	2.83 (0.98)	2.65 (1.05)	2.57 (1.17)	2.29 (0.95)	3.52 (1.05)	78.8 (20.9)

There was a notable difference in scores between males and females, with males reporting higher scores for all domains except “inclusion and safety”. Intriguingly, both genders reflected a similar pattern for reporting the highest score for physical space and the lowest for inclusion and safety. However, four domains showed statistically significant associations at the 0.05 level: peer community, faculty relationships, academic climate, and meaningful engagement.

Among the different academic year levels, first-year students reported the highest score for community of peers (3.64 ± 0.88) and the lowest for inclusion and safety (1.82 ± 0.90). Similarly, second- to fourth-year students reported the highest scores for physical space and the lowest scores for inclusion and safety. As the academic year progressed, fifth-year students and interns obtained the lowest scores in meaningful engagement (2.26 ± 0.94 and 2.18 ± 0.95, respectively), but the highest score was given for physical space among 5th-year students (3.42 ± 0.93) and communities of peers for interns (3.43 ± 0.87). There were statistically significant associations with all subscales except physical space (*P* value = 0.33).

Students with high GPA (4.50–5.0) recorded higher results across all domains than did their peers who earned a lower GPA (i.e., less than 4.00), with the exception of inclusion and safety. It is interesting to note that the physical space domain stood out as the highest scorer for all groups, while the scores for inclusion and safety fell short among all groups according to GPA. There was a statistically significant association with the first three domains only, community of peers, faculty relationships, and academic climate.

Employed students reported higher scores on measures related to community engagement reflected in the community of peers (3.70 ± 0.63), while unemployed and freelance students had the highest scores for physical space (3.52 ± 0.95 and 3.65 ± 1.13, respectively). The inclusion and safety subscale scores were the lowest for unemployed and employed students (2.25 ± 0.95 and 2.49 ± 1.20, respectively), while freelancers reported the lowest score for the academic climate subscale (2.36 ± 0.61). Employment status was significantly associated with only the mentoring subscale (*P* value = 0.02).

Students who were single attained the highest average score of 3.37 ± 0.91 on the physical space domain, while inclusion and safety presented a challenging component (2.26 ± 0.95). Conversely, those who were married or engaged garnered the highest community of peer ratings, averaging 3.53 ± 0.78, and the lowest for faculty relationships, with a mean value of 2.72 ± 0.92. Students residing with family or in private accommodations, as well as those with families living in Riyadh or outside Riyadh, reported the highest scores in the physical space domain and the lowest scores in inclusion and safety. However, the association was not statistically significant between all groups or across all subscales (*P* value > 0.05)*.*

## Qualitative results

Participants of both genders, senior and junior years, represented the FGDs (Table 4). One participant was employed, and all were living with their families.

**Table 4:** Demographic data of the focus group participants

Participants	Year	GPA	Gender	Age
P1	4th	4.60	Female	22
P2	5th	4.76	Male	23
P3	Intern	3.92	Male	24
P4	4th	4.57	Female	22
P5	Intern	3.05	Male	27
P6	1st	4.98	Male	19
P7	4th	4.50	Female	22
P8	3rd	4.90	Female	22
P9	4th	4.76	Male	23
P10	5th	4.90	Female	23
P11	4th	4.84	Male	22
P12	1st	4.95	Female	19

As open-ended questions were used to collect data, themes were derived from deductive and inductive analysis. Inductive analysis was based on a priori themes based on the JHLES domains. Table 5 shows the domains in which participants’ perceptions were compared with the quantitative findings. Some qualitative findings aligned with the quantitative findings, while others contradicted or explained them.

**Table 5:** Joint display of the quantitative and qualitative interpretation and deductive analysis of focus groups

Section I: Inductive analysis		
**JHLES Domains**	**Quantitative**	**Qualitative interpretation**
Community of peers	In this domain, males had higher average score than females Students with higher GPA had higher average scores 1st year and intern had higher average scores than other academic years	Most participants thought that friends and colleagues from the college were the only source of support they have: *“My friends, my inner circle, are my study groups. We help each other to move forward”* P1 Three participants believed that friendship can also be a source of stress if their friends themselves were. One participant shared her experience and said: *“When I was in my first year, I was so stressed and emotionally destroyed because of the anxious friends around me” P8*
Faculty relationships	In this domain, males had higher average score than females The highest average score was in the 1st year and decreased with the following years Almost similar average score among the three groups classified by their GPA	Most participants agreed that most doctors were neutral, not encouraging nor discouraging. Although doctors who take one of the stans have high influence on the students Participants think that there are a small number of faculty who are genuinely supportive. One participant mentioned: “*I struggled a lot during my journey. One of the teaching physicians knew about this and he was sending me emails to see if I’m doing okay or advise me on what to do as much as he could just from the kindness of his heart*” P5 “*It differs when you hear supporting words from a faculty than from a colleague. The doctor has already finished the journey, they know better*” P9 Participants did not appreciate the way some faculty think they are supporting them. A fifth-year student mentioned: *“One doctor told us in my first day in the college: ‘if you did not study for 4 h a day you will fail’…I’m not sure if he was motivating us but it was so stressful to do that” P2* *“The environment within the class is stressful. We can’t ask a question freely without being judged” P1*
Academic climate	Males had higher average score than females The highest average score was in the 1st year and decreased with the following years Almost similar average score among students with GPA 4.0 or more, but less with GPA lower than 4.0	faculty were described by participants using words such as “*arrogant*”, “*disrespectful*” and “*they are always right!*” One participant who was a group leader, mentioned: “*They say ‘send an email!’ and they do not reply to my email*” *P11*
Meaningful engagement	Males had higher average score than females The highest average score was in the 1st year and decreased with the following years Almost similar average score among students with GPA 4.0 or more, but less with GPA lower than 4.0	Participant did not believe in the support services provided to them from the college. For example: “*students’ council is just a name to tick the checklist…they do not have power to change. The reports they produce contain the same complaints for three years!*” *P3* Only one student mentioned a positive story with the students’ council of the college, yet he believes that the council leader, at that time, was active and helpful which are not always characteristics of the council members Participants believed that there is no system to support them: *“I failed internal medicine 3 times and I went to the department to talk to someone but each time I was referred to someone else in the department and still, no one helped me or listened to me” P5* However, on the other hand, if they had technical issues collectively such as, delays in responding to their request to activate their cards, responses are usually quick from the college administration
Mentoring	Males had higher average score than females The average score increases with years to reach the highest at internship with a decline during 4th year Almost similar average score among students with GPA 4.0 or more, but less with GPA lower than 4.0	Only one participant had a previous experience with his mentor in the college. He mentioned: *“I contacted my mentor and he said ‘why are you so into fixing this? you would be lucky if you get a job in a remote village…after that I just stopped contacting him” P5* Another participant noted: *“We can find knowledge everywhere, but we need guidance, example, character from doctors that we look up to” P11*
Inclusion and safety	Almost similar average score however, slightly higher among females The average score increases with academic years where the highest was during 5th year and internship Almost similar average score among the three groups classified by their GPA	Regarding gender roles in the learning environment, each gender group perceived that they are discriminated against. Male participants felt the following: *“Doctors treat us harder. They give us lower grades for the same exams, the give us more responsibilities during internship” P3* On the other hand, female participants thought: *“Males have advantage as they can build relationship with male doctors, who forms the majority of the doctors in the main medical specialties, thus get more research opportunities” P4* Apparently, all participants experienced or still experience stress: *“When I was in my first year, I was so stressed and emotionally destroyed because of the anxious friends who were around me” P8* *“The environment within the class is stressful. We can’t ask a question freely without being judged” P1* *“High expectations make us stressed to an extent that your best is never enough” P5*
Physical space	Almost similar average score with slightly higher among males The highest average score among 1st year students and the lowest among interns, but almost similar in between Almost similar average score among students with GPA 4.0 or more, but less with GPA lower than 4.0	As the college buildings are relatively new and all participants in the focus group discussions were living with their families, physical space domain as part of JHLES score was not a concern to the participants. Thus, the discussion may not reflect the physical space in the university housing as a possible factor that could affect learning
Section II: Deductive analysis		
Theme	Subtheme	Qualitative interpretation
Perceptions about studying Medicine at KSU	Proudness	Despite the stress felt by the participants as a result of high expectations from graduates from a well-known University, they still documented feeling “proud” and “honored” because they believed that being accepted in the college of medicine at this University is a challenge that proves that they are “the best” The proudness come from challenges students face during their journey, *“I am proud of myself, I went through a lot, it’s a tough college” P5* The reputation of the University, *“The legacy of this University and most of the excellent Saudi doctors are graduates from KSU” P4* And getting acceptance in the college is a self-prove that they are “the best”, *“More than 2000 male students have applied to this college at KSU, but only 200 were accepted…they only take the best” P6* On the other hand, participants thought that the environment of the college is toward resisting rapid development due to the presence of older physicians. He explained: *“Old and experienced physicians work here but they are not keen anymore to change even to something better, they even resist the change” P5*
	High expectations and competition	According to the participants, high expectations from their families and faculty doctors, negatively influence the study experiences and put more stress on students *“High expectations make us stressed to an extent that your best is never enough” P5* High expectations made the study environment highly competitive among students. One participant summarized situations where competition can be prevalent between students: *“There are three kinds of competition among students: on improving CVs by volunteering and leadership experiences without telling their friends about opportunities for this, knowledge by showing it in front of the doctors which makes others feel bad, and grades” P6* Competitiveness was attributed to high ambitions of medical students: *“You do not see a medical student in KSU without high ambitions” P5* It was also attributed to the pressure put on students by the doctors: *“The competition among students is a result of doctors comparing students with each other. One time a doctor asked me, sarcastically, in front of the class about my GPA just because I hesitated in answering a medical question he posed” P4* About the effect of these experiences, positive consequences such as motivation to study more, can be experienced despite the strong negative emotional effect. One participant mentioned: *“It pushes you forward but it gets extremely overwhelming with time it consumes you” P5*
	Views about the curriculum	Although, participants believed that some of their comments on surveys about courses were heard as they see the changes throughout the years. In addition, this makes some students taking course evaluation seriously however, this does not positively change students view about the curriculum as one explained: “*Yes we see some changes that were based on our comments but the curriculum needs fundamental changes*” P5 Only one participant thought that the contents of the curriculum is “*good*” but he would suggest changes in: “*Reorder of the lectures, timing distribution among blocks and credit hours*” P3 Words like “*bad*”, “*I don’t think a curriculum exist!*”, and “*random*” were some of the comments mentioned by the participants. One participant explained: “*Lectures do not connect logically, each doctor gives a session from his own experience in practice, there is no one reference that they can agree on for students’ level*” P5 He added: “*I feel that they are challenging us rather than try to make us learn and understand*” P5

## Discussion

Evaluating the learning environment for medical students is essential for improving their professional standards, knowledge, and skills. This mixed methods study explored medical students' perceptions about the learning environment at the College of Medicine, a well-known university in Saudi Arabia, King Saud University. This study is two-pronged, first, to quantitatively assess students’ perceptions of the COM-KSU learning environment and, second, to qualitatively explore their experience in the same medical school.

Our study yielded an overall average score of 81 out of 140 on the JHLES. Notably, there was no predefined threshold for a passing or positive score on this tool. Compared to the original study where the scale was first used and validated, the average score in our study was lower (107 vs 81, respectively) [7]. This discrepancy might be related to the original study's single-institute design affects the generalizability of its results, and the differences in student characteristics due to the U.S. requiring a bachelor’s degree for medical school admission, unlike KSA, where students enter directly after high school, play a role. Additionally, the original study did not focus on the "hidden curriculum" influenced by organizational culture and structure, which may explain the discrepancy given the distinct social, organizational, and learning cultures between our context and the American one. However, our results were consistent with those of other studies that were conducted in other medical schools in different countries, including Malaysia, India and Pakistan, ranging from 81.1 to 86 [9–11].

Two previously published studies in the same setting, COM-KSU (2008 and 2017), utilized the DREEM survey and revealed that medical students reported different average scores (89.9 out of 200 and 171.57 out of 250, respectively) [5, 6]. Compared to the current study utilizing the JHLES, we may compare the findings based on a significant correlation between the two measures that support the use of the JHLES in the assessment of the same construct [11]. This comparison yielded reassuring results that the perceptions of medical students are still positive, with variations in the domains of LE, as described below. The added value of the qualitative component of the current study elicits more depth in understanding LE in the COM-KSU.

Although there was no difference among male and female students in the DREEM overall average score in a previous study that was conducted at the same college in 2017, our study revealed a higher overall average score among males (83.4) than females (77.5). The lower recorded score among females might be explained by their tendency to have higher expectations of a learning environment that was not achieved as their counterpart expected [12, 13]. For explanations, male students had higher scores in different domains related to their relationships with the faculty and peers, including mentorship, peer support, and the academic climate. Nevertheless, both genders perceived a negative view where they expressed potential gender discrimination in the focus group interviews. Male students felt that they were treated differently than females, while their counterparts believed that males had more opportunities to build relationships with the faculty and gain more experience accordingly.

In terms of academic years, the domains and overall average scores decreased as the students progressed from the first year to their internships, with an exceptional decrease in the third year followed by the recovery of scores afterward. Nevertheless, students in the first year had higher average scores than interns, possibly due to the new environment and the support provided during their first year. Qualitative group interviews elaborated more on this variation, where medical students in the first year felt a sense of pride and honor upon being accepted in the COM-KSU. They believe that this was a validation of their social status.

Although the relationship between medical students’ feelings of pride in belonging to their college and the learning environment is complex and multifaceted [14], a positive and supportive learning environment that fosters a sense of belonging can enhance medical students’ feelings of pride and affiliation with their college [15, 16], which is evident among first-year medical students. In contrast, a negative learning environment that lacks support and inclusivity can detrimentally impact medical students’ feelings of pride and belonging [17]. Nevertheless, first-year students still experienced negative emotional effects that were not captured by the quantitative questionnaire due to the lack of professional identification they encountered when they moved from the preparatory year to medical school.

However, the decrease in the average score during the third year could be explained by engagement in clinical rotations and practical applications instead of merely learning basic science. This perception was explained during focus group interviews where students explained the third year as the most challenging due to the preparation for their actual medical practice. This included starting to see patients, taking medical history, and performing physical examinations. Interestingly, this result was consistent with other studies that were conducted in different medical schools, although different assessment tools were used, including MSLES, DREEM, and the same tool used in this study (i.e., JHLES) [3, 7, 10]. In contrast, other studies have shown that medical students feel more satisfied with clinical practice than with basic science during the first and second years [12, 18, 19].

This paradox might be explained by the difficulty students faced at the beginning of the clinical year, after which it decreased or diminished after they gained confidence in their practice under the supervision of well-trained faculty [20–22]. Hence, higher average scores in the following years could be explained by the maturity of the medical students and their ability to overcome early difficulties after they have more experience during clinical rotations. In the COM-KSU, medical students in their fifth year are prepared to experience life as physicians where they have pure clinical experience joining medical teams, attending rounds, clinics and doing procedures under the supervision of trained faculty and senior doctors. Hence, when mentoring was assessed among medical students, their perception reflected by the average score given to this domain increased as the number of academic years increased, with the highest score occurring during the internship. Mentorship plays an important role in the learning environment, as described in other studies [23–25]. The importance of the student‒faculty relationship and the enhancement of faculty influence on students are supported by the qualitative findings, which demonstrate that students' perceptions of faculty support vary, which is congruent with other studies [26–28]. However, a study revealed that the majority of faculty members are not prepared to provide the kind of support that has been shown to be most effective for students [29].

Furthermore, the meaningful engagement of students declines as the academic year progresses, as expressed by students’ responses to this domain in the JHLES. The qualitative approach elaborated more when students complained about the lack of support provided by the student council, which the COM-KSU perceived as the hub where medical students can engage and obtain the required support. From the students’ perspective, the student council was not able to provide effective support or bring about significant changes for students facing challenges related to their medical study needs. The qualitative study participants agreed with the findings of other local studies, highlighting the absence of a supportive environment for students in our local colleges [12, 30, 31]. On the other hand, the majority of students reflected positively on peer support, where they found it to have a positive impact on them. They identified college friends and colleagues as the main sources of support, which was congruent with other studies that explained the same attitude [32–34].

According to the students’ performance measured by their GPA, students with higher GPA had higher JHLES scores, both overall and domain average scores. High-achieving students tend to have more positive perceptions of the learning environment than do students with lower GPAs [10, 11, 21, 22, 35]. This could suggest a positive association between academic achievement and students’ perceptions of the educational setting [18, 19, 36–38]. However, students experienced positive consequences from high competition in the learning environment due to family and physician expectations that were captured during the focus group discussion. Similar results were found in another study that was conducted in the medical school of the University of Valladolid [39].

Inclusion and safety were negatively perceived in this study among medical students at all levels, regardless of their gender, academic year, or performance, which was reflected in their GPAs. This finding was consistent with other studies measuring the same domain average score of Cyberjaya University College of Medical Sciences (CUCMS), Nil Ratan Sircar Medical College (NRSMC), and College of Medicine and Sagore Dutta Hospital (CMSDH) [9, 11]; however, this finding was in contrast to that of PUGSOM [40]. A possible explanation might be related to the aforementioned reasons, which were associated with students’ perceptions of gender discrimination, stress in the first year due to the new environment and in the third year due to engagement in clinical practice, and their achievements, which elevated stress when they had lower GPAs. Previous studies have shown that the prevalence of stress is greater during the first three years of medical education, which is consistent with our findings [35].

In contrast, the physical space domain in our study received the highest score, where we believe that physical space has improved as a result of the college's 2018 expansion [41].

## Strengths and limitations

One key strength of this study is the employment of a comprehensive mixed methods approach to gain an understanding of how students perceive their learning environment. This approach collects numerical data, delves deeply into the students’ experiences and feelings, and provides valuable insights through the integration of findings from both approaches. Another strength of this study is the large number of participants from different academic years, which allows for a diverse range of perspectives from both new and experienced students.

Nevertheless, convenience sampling may not fully represent the student population and limits the generalizability of the findings. Additionally, focusing on one institution may not capture the experiences of students across different settings, cultures, or cities, potentially limiting the applicability of any recommendations to other medical colleges or regions. However, the large sample size, the diversity of data and the integration of results may enhance the transferability of the findings.

## Recommendations for educational institutions

1. Enhance faculty development: Address the issues of perceived neutrality and reported negative interactions with faculty by investing in faculty development programs. These programs should focus on improving communication skills and mentoring abilities and cultivating more supportive and encouraging faculty‒student relationships. Creating opportunities for regular feedback from students can also aid in faculty improvement. This is important as students showed high tendency to be influenced by advice from faculty member.
2. Cultivate Supportive Environments: Foster a less stressful academic climate by promoting a culture of mutual respect and collaboration within the institution. Encourage open dialog between students and faculty, where questions and concerns can be raised without judgment. Stress management and well-being programs should be implemented to help students cope with academic pressures.
3. Revise Curriculum and Mentorship Programs: Address curriculum concerns by engaging students in the curriculum development process. Consider their suggestions for better organization, logical flow, and references. Additionally, structured mentorship programs that connect students with experienced doctors who can provide guidance, share experiences, and serve as positive role models should be established.
4. Evaluate and Improve Support Services: Reevaluate the effectiveness of support services such as the students' council and academic support departments. These services are responsive to students' needs and have the authority to enact meaningful changes. Regularly solicit feedback from students to gauge the impact of these services.
5. Promote Inclusivity and Gender Equity: as FGDs showed that both genders feel discriminated against, creating initiatives to address perceptions of discrimination and gender bias within the learning environment is important. This may involve raising awareness, offering training on gender sensitivity, and implementing policies that promote inclusivity and equal opportunities for all students, regardless of gender.

## Recommendations for further research:

1. Longitudinal studies should be conducted to track the changes in students’ perceptions and experiences. This will help us identify emerging trends and understand the long-term effects of interventions and policy changes.
2. This research can be expanded by including studies with medical schools or institutions to validate our findings and assess how applicable they are in diverse educational settings.
3. The use of mixed methods research in the field of education should be further explored. Investigate approaches that combine qualitative and deductive methods to gain deeper insights into students’ educational experiences.
4. Dive deeper into specific areas highlighted in this research, such as mentoring programs and concerns related to the curriculum. Explore ways to enhance mentoring effectiveness and develop strategies for improving the curriculum to create a learning environment.
5. Interventions targeted at addressing identified areas should be implemented for improvement while thoroughly evaluating their impact. This will enable institutions to assess the effectiveness of these interventions based on data-driven decisions leading to the enhancement of education.

## Conclusion

This study was the first to assess the learning environment of medical students at COM-KSU through quantitative and qualitative approaches. The overall average JHLES score indicated room for improvement, in line with global trends. Gender disparities, challenges in different academic years, and the critical role of mentorship were identified. Academic performance correlated positively with students' perceptions, while inclusion and safety were areas of concern. The physical space domain received the highest score, reflecting investments in infrastructure. These findings underscore the need for targeted interventions to address gender disparities, enhance mentorship, improve student engagement, and ensure inclusivity and safety, ultimately enhancing the educational experience of COM-KSU medical students.

## Data Availability

The datasets used and analyzed during the current study are available for request from the corresponding author.

## Abbreviations

LA: Learning environment
JHLES: Johns Hopkins Learning Environment Scale
COM-KSU: College of Medicine at King Saud University
DREEM: Dundee Ready Educational Environment Measure
FGDs: Focused group discussions
MSLES: Medical School Learning Environment Survey
CUCMS: Cyberjaya University College of Medical Sciences
NRSMC: Nil Ratan Sircar Medical College
CMSDH: College of Medicine and Sagore Dutta Hospital
